# Quantitative Proteomics Analysis Reveals Novel Targets of miR-21 in Zebrafish Embryos

**DOI:** 10.1038/s41598-017-04166-x

**Published:** 2017-06-22

**Authors:** Ying Wu, Qi-Yong Lou, Feng Ge, Qian Xiong

**Affiliations:** 1 Key Laboratory of Algal Biology, Institute of Hydrobiology, Chinese Academy of Sciences, Wuhan, 430072 China; 2 University of Chinese Academy of Sciences, Beijing, 100049 China

## Abstract

MicroRNAs (miRNAs) are noncoding RNAs which control gene expression by the suppression of translation or the degradation of mRNAs. Dre-miR-21 (miR-21) has been reported to impact cardiac valvulogenesis in zebrafish embryos. However, the target genes of miR-21 are still largely unknown. Here a tandem isobaric mass tag (TMT)-based quantitative proteomic strategy was employed to identify the global profile of miR-21-regulated proteins. A total of 251 proteins were dysregulated after miR-21 knockdown, suggesting that they may be regulated by miR-21. Bioinformatics analysis indicated that these differentially expressed proteins (DEPs) participate in various biological processes, suggesting that miR-21 may be involved in diverse cellular pathways. Sixteen DEPs were also predicted to be miR-21 targets by at least two algorithms, and several candidate target genes were selected for further luciferase reporter analysis. The results showed that genes encoding tropomyosin 1 (*tpm1*) and poly(rC) binding protein 2 (*pcbp2*) are direct miR-21 targets. Taken together, our results not only reveal a large number of novel miR-21 regulated proteins that possess pleiotropic functions, but also provide novel insights into the molecular mechanisms of miR-21 regulation of zebrafish cardiac valvulogenesis and embryonic development.

## Introduction

MicroRNAs (miRNAs) are a class of small noncoding RNAs which regulate gene expression post-transcriptionally. A single miRNA may regulate multiple genes in diverse cellular processes by binding to complementary sequences on target mRNAs, leading to the repressed expression or degradation of mRNAs at the translational level^1^. miRNAs can greatly influence gene expression, as more than 60% of human protein coding genes can be regulated by miRNAs^2^. The expression patterns of over 100 miRNAs have been characterized in zebrafish^3^. Additionally, several miRNAs are known to regulate cardiogenesis^4–7^. For example, during the early stages of mesoderm formation, miR-142-3p plays a crucial regulatory role in cardiogenesis, hematopoiesis and somitogenesis^6^. Cardiogenesis is regulated by miR-143-induced suppression of retinoic acid signalling^4^. miR-1 regulates the balance between proliferation and differentiation during cardiogenesis by targeting key cardiac regulatory proteins^5^. Two recent studies revealed that miR-21 is also crucial in the regulation of cardiac valvulogenesis^7, 8^.

miR-21 is conserved between humans and zebrafish, with only two base pair differences in their mature forms. In zebrafish, miR-21 is specifically expressed in the heart and brain, and it seems to be confined to the valvular endothelium within the heart^3^. In mammals, miR-21 is reported to be highly expressed in malignant tissues and thought to play important roles in tumour invasion^8, 9^. miR-21 expression is also detected during cardiac remodelling, and it has been implicated in heart failure and several forms of cardiac stress^10–13^. Several target genes of miR-21 have been identified in humans, including tumour suppressor programmed cell death 4 (*PDCD4*)^14, 15^, phosphatase and tensin homologue tumour suppressor (*PTEN*)^16^, tropomyosin 1 (*TPM1*)^17^ and sprouty RTK signalling antagonist 2 (*SPRY2*)^18^. In zebrafish, miR-21 is critical in heart valve formation. It plays critical roles in valvulogenesis by regulating the same targets as those of the human/mouse miR-21 (*pdcd4*, *sprouty* and *ptenb*)^7^.

However, only a few miR-21 targets have been validated, and their functional mechanisms are largely unknown. miRNAs may regulate gene expression by suppression translation without mRNA degradation^19, 20^. Thus, determining the expression levels of mRNAs is not ideal for identifying miRNA targets, as mRNA levels do not necessarily correlate with protein levels^21–23^. On the other hand, the discordance between the transcriptome and proteome can be increased due to the different half-lives of mRNAs and proteins. Furthermore, the efficiency of miRNA-mediated gene silencing can be decreased by poly(ADP-ribosyl) ation of Argonaute/RNA-induced silencing complex (RISC), implying that the presence of miRNA alone may not lead to reduced protein expression^24^. Therefore, mRNA abundance is not the best surrogate indicator for the biological activity of those proteins.

To systematically discover the regulated proteins of a miRNA, high-throughput mass spectrometry (MS)-based proteomic approaches are a very efficient and effective way to fully elucidate their functional mechanisms. In a previous study, we successfully identified miR-21 targets in human cancer cells by employing a Stable Isotope Labelling by Amino acids in Cell culture (SILAC)-based quantitative proteomic approach^25^. In the present study, global proteomic profiling was carried out to identify targets of miR-21 in zebrafish embryos. Using a Tandem Isobaric Mass Tag (TMT)-based quantitative proteomic strategy, we found that 251 out of 2675 proteins quantitated were differentially expressed after miR-21 inhibition. Our results show that miR-21 can regulate numerous cellular pathways by modulating the expression of hundreds of proteins. We further confirmed that tropomyosin 1 (*tpm1*) and poly(rC) binding protein 2 (*pcbp2*) identified from a proteomic profiling are direct targets of miR-21 using luciferase assays.

## Results

### Effect of miR-21 Knockdown

**Figure 1:**
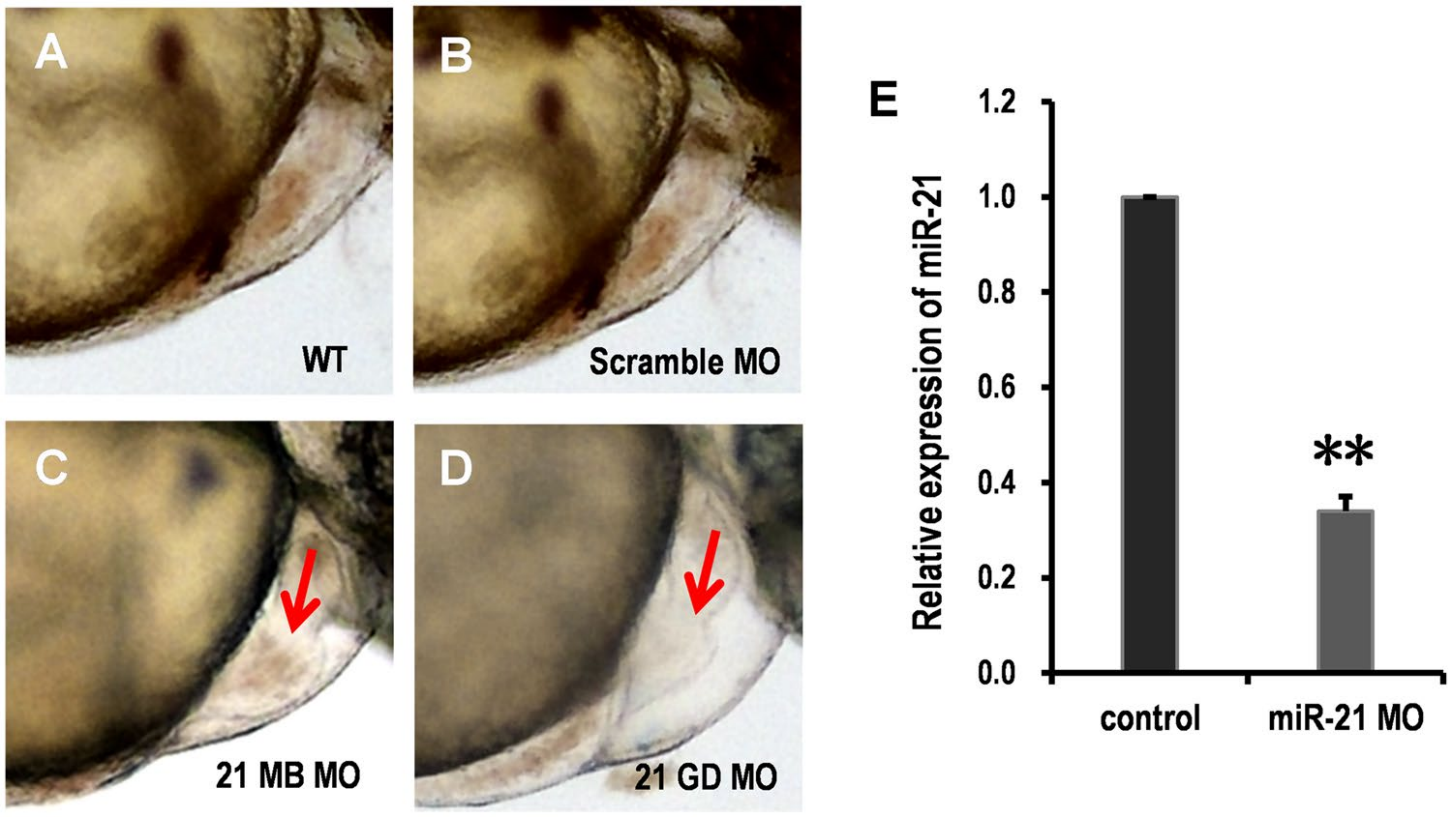
The effect of miR-21 knockdown on the zebrafish valve development. (**A**–**D**) Both wild-type and scramble MO control-injected embryos show normal AV ring constriction ((**A**,**B**) arrow) at 55 hpf, whereas miR-21 knockdown results in loss of the normal AV constriction ((**C**,**D**) arrow) (**E**) Relative expression of the mature miR-21 was measured by qRT-PCR using RNA isolated from the whole bodies. Injection of miR-21 MOs knocked down the expression of miR-21 to nearly 30%. **P < 0.01.

In zebrafish, the cardiac expression of miR-21 was first detected at 48 hours post fertilization (hpf), and was restricted to the atrioventricular valve (AV) ring endocardium^26^. Then, miR-21 expression was observed in the cardiac outflow tract and the developing central nervous system by 72 hpf^26^. Two genomic copies of *miR*-*21* exist in zebrafish, which can generate the same mature miRNA (miR-21-1 and miR-21-2). In agreement with previous studies, injection morpholino oligonucleotides (MOs) targeting pre-miR-21-1 at a high dose (up to 40 ng) did not cause any consistent cardiac phenotype. However, injection of only 4 ng of a MO targeting pre-miR-21-2 (miR-21-2, multi-blocking) resulted in incomplete looping of the heart tube in 80% (100/120) of the embryos (Fig. 1). These phenotypes are highly consistent with those observed in previous studies^26^. MOs targeting miR-21-2 was used in the miR-21 knockdown in quantitative proteomic strategy.

### Screening of Potential miR-21-regulated Proteins Using a Quantitative Proteomic Strategy

**Figure 2:**
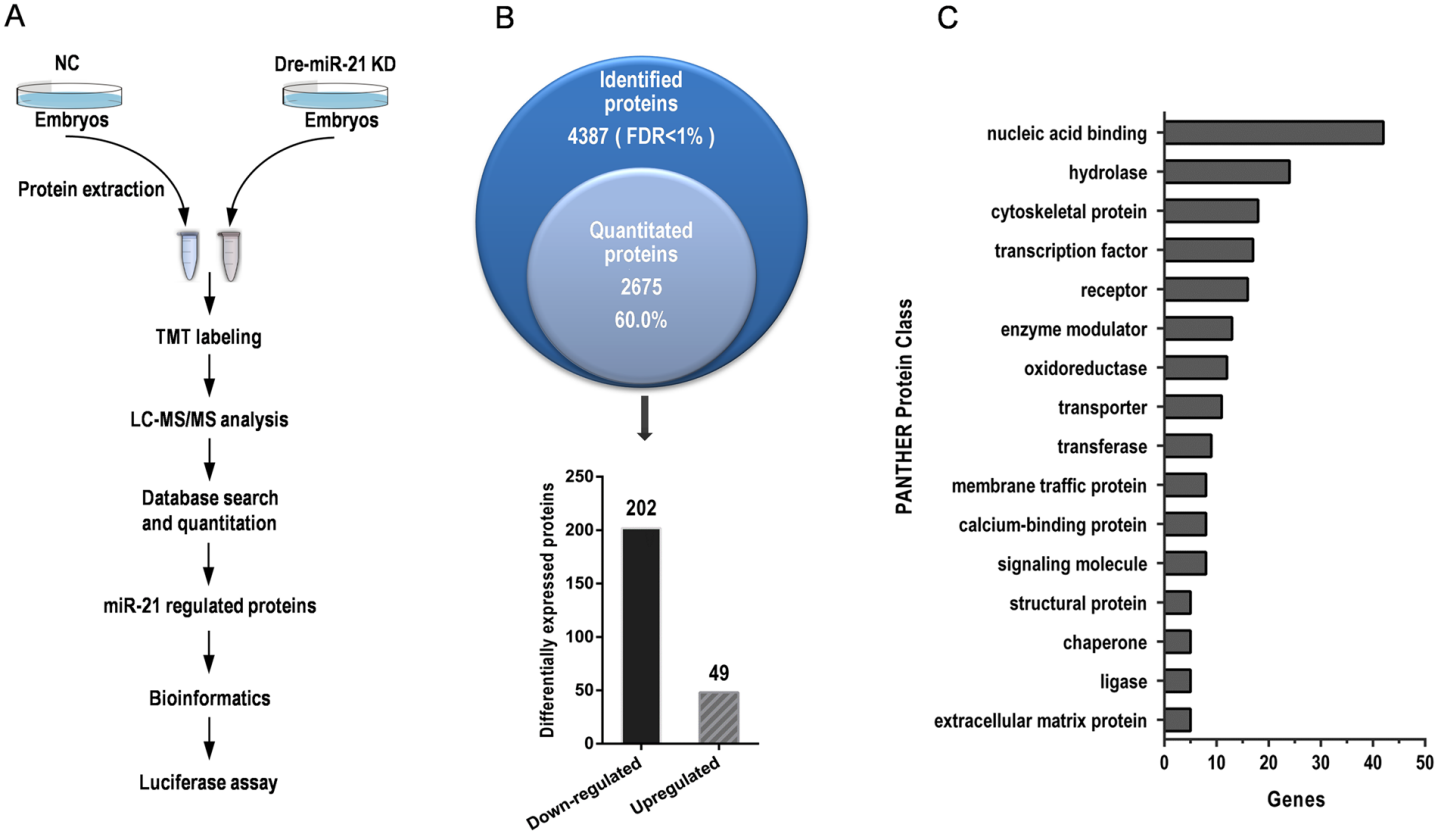
Quantitative proteomic identification of miR-21-regulated proteins in zebrafish embryos. (**A**) Workflow for the identification of miR-21-regulated proteins. (**B**) Overview of the proteomic results. (**C**) PANTHER Protein Class ontology classification of the 251 proteins differentially expressed after miR-21 knockdown.

To identify potential miR-21 target genes, we performed TMT-based quantitative proteomic analysis as summarized in Fig. 2A. A total of 4387 proteins were identified, of which 2675 proteins (Table S1) and 20,174 unique peptides were quantified in our experiment at 1% false discovery rate (FDR). The overview of the proteomic results such as protein mass distribution, peptide distribution and length of peptides are presented in Fig. S1. The majority of the quantitated proteins had a log2 fold change (miR-21-knockdown group (KD)/negative control group (NC)) between −0.27 and 0.27 (Fig. S2). Two hundred and fifty-one proteins with a log2 fold change ≥0.27 or ≤−0.27 (CV < 30%) were considered to be dysregulated after miR-21 inhibition (Table S2). Among these DEPs, 49 were upregulated and 202 were downregulated after miR-21 knockdown.

### Bioinformatics Analysis of miR-21-regulated Proteins

**Figure 3:**
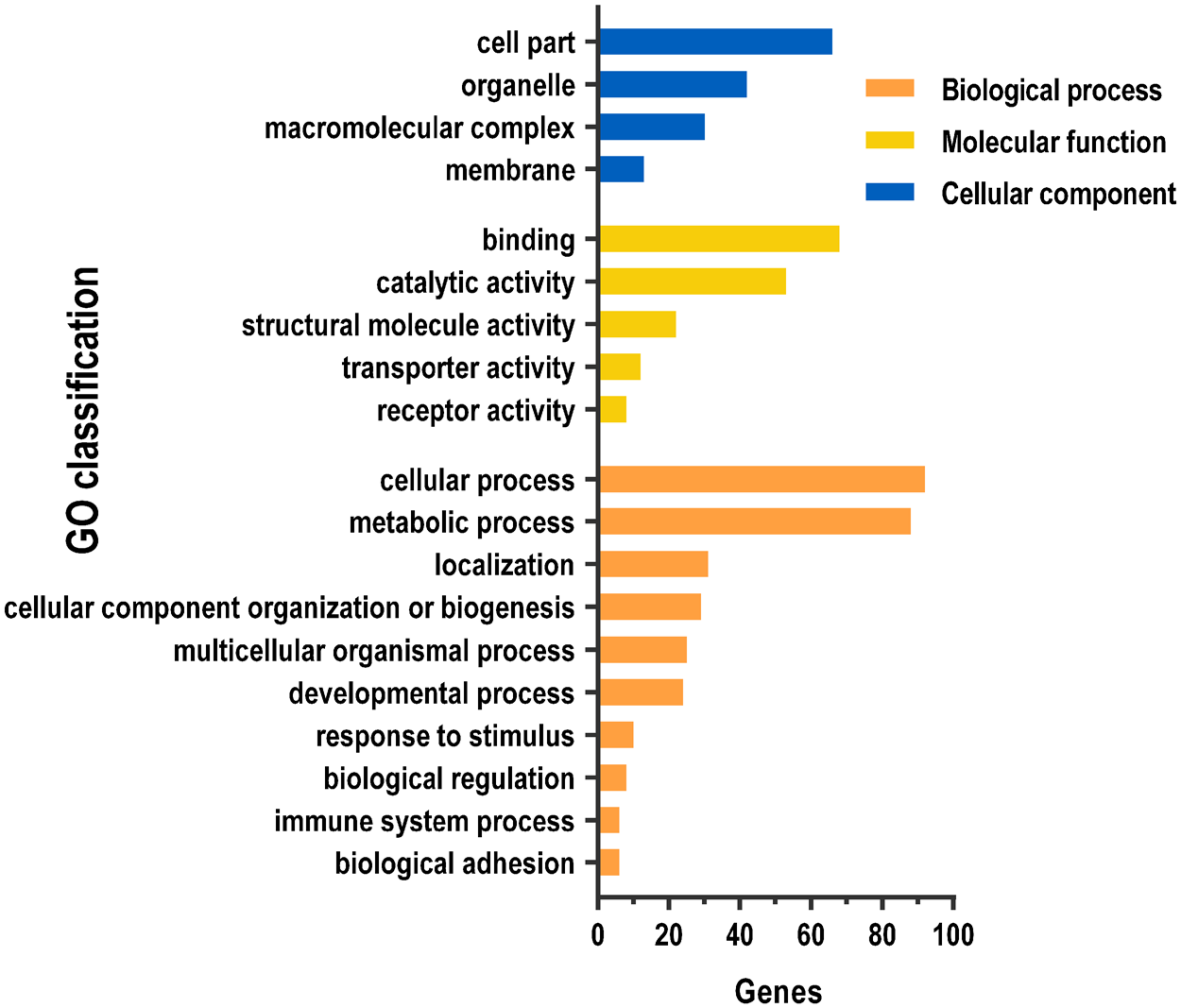
GO classification of miR-21-regulated proteins. GO distribution of miR-21- regulated proteins according to their biological process, molecular function and cellular component categories. Categorization was based on information provided by PANTHER.

To gain insights into the biological functions of miR-21, we first classified the DEPs using Protein Analysis Through Evolutionary Relationships (PANTHER) Protein Class ontology (http://www.pantherdb.org/). The 251 proteins were classified into 17 classes (Fig. 2C), the largest five of which were ‘nucleic acid binding’, ‘hydrolase’, ‘cytoskeletal protein’, ‘transcription factor’ and ‘receptor’. We next analysed the Gene Ontology (GO) distribution of the DEPs. The GO biological process classification results revealed that many DEPs were assigned to ‘metabolic process’, ‘localization’, ‘multicellular organismal process’ and ‘developmental process’ (Fig. 3). Panther pathway classification revealed that the DEPs are involved in various pathways, including the Integrin and Wnt signalling pathways (Table S3). Taken together, these results imply that miR-21 may be an important regulator of diverse cellular functions across widespread biological processes during zebrafish embryonic development. A PPI network of the DEPs and two reported miR-21 targets (*pdcd4* and *spry2*) were generated using the Search Tool for the Retrieval of Interacting Genes/Proteins (STRING) database (version 10.0, http://www.string-db.org/), revealing that 125/251 DEPs were involved in a PPI network (Fig. S3). The DEPs also interacted with validated miR-21 targets in the zebrafish (Fig. S3).

### miR-21 Target Prediction

**Figure 4:**
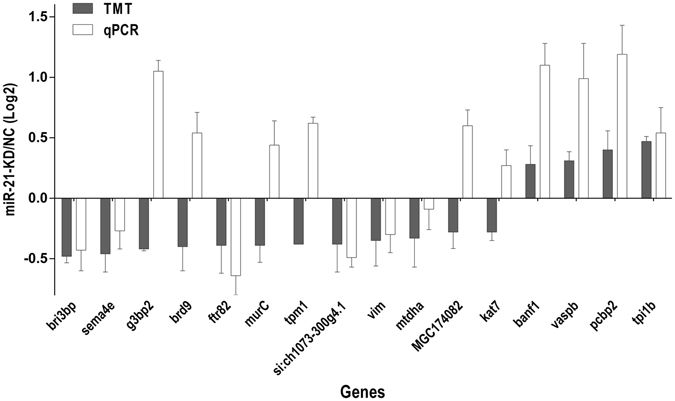
The mRNA expression level of the DEPs as detected by qRT-PCR. Comparison of log2 expression of 16 selected DEPs measured by TMT proteomic analysis and qPCR. Positive and negative log2 expression ratios represent up- and down-regulation after miR-21 Knockdown respectively.

Six different algorithms were employed to predict the targets of miR-21 as described above. The mRNAs of 16 DEPs were also predicted to be miR-21 targets by at least two algorithms (Table S4). TMT quantitative proteomic results showed that 12 genes were downregulated and 4 genes were upregulated after miR-21 knockdown. These genes are potential miR-21 targets. We next set out to determine whether the mRNA expression level of these DEPs also changed by using real-time quantitative reverse transcription PCR (qRT-PCR), and the results showed that the expression of 10 DEPs was consistent with their mRNA levels (Fig. 4). However, six downregulated DEPs were detected to be significantly increased at the mRNA level (Fig. 4), which may due to the lack of synchrony between mRNA and protein expression or other post-transcriptional regulation mechanisms. Thus, it is essential to identify miR-21-regulated targets at the protein level.

### Validation of the proteomic data

**Figure 5:**
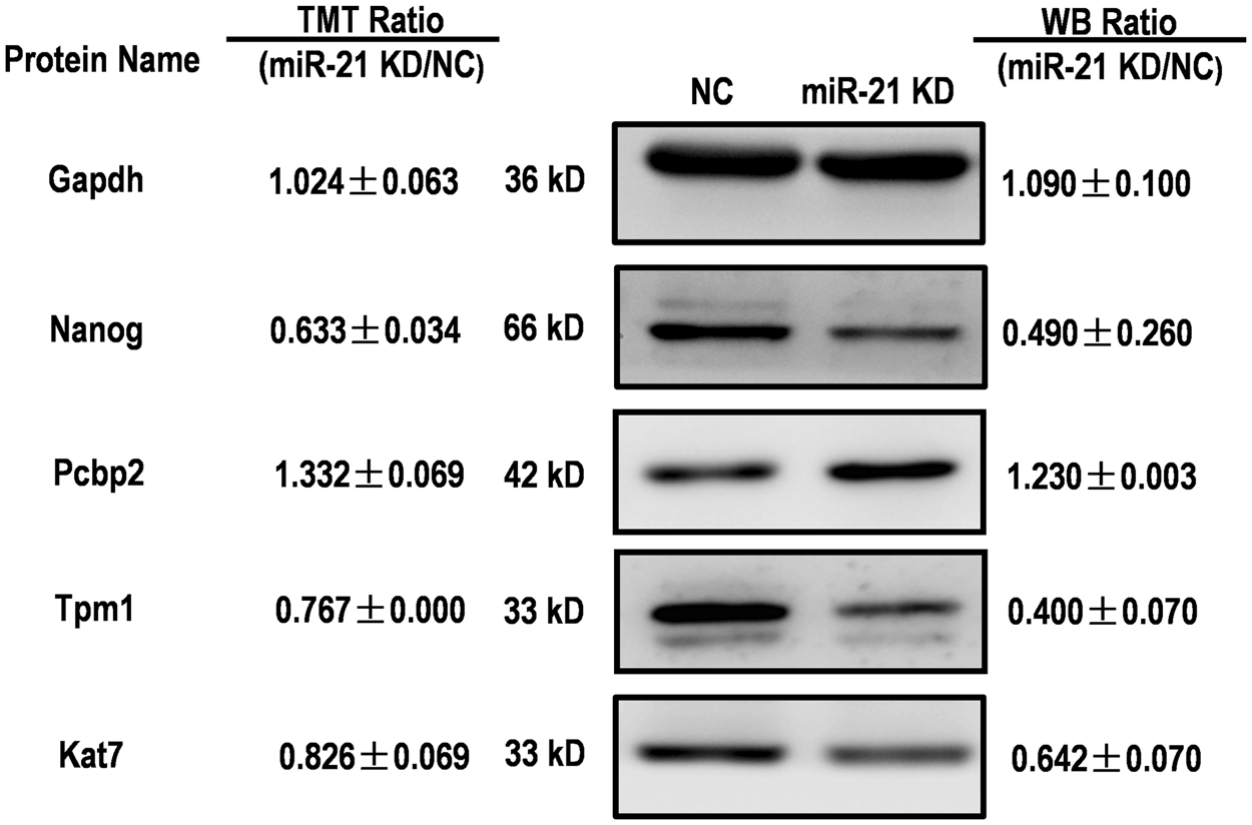
Validation of the proteome data. Western blot analysis of protein expression levels of four representative proteins quantified by proteomics after miR-21 knockdown. Representative Western blot of three (n = 3) independent experiments are shown. Full image of the Western blots is shown in Fig. S4. Fold changes of protein levels were determined using Image J software and normalized by protein concentration.

Several DEPs of biological interest (nanog homeobox (Nanog), Pcbp2, Tpm1 and Histone acetyltransferase (Kat7)) from the 251 proteins dysregulated after miR-21 knockdown were further validated by WB (Fig. 5). The WB results demonstrated that the expression of these proteins was consistent with the TMT quantitative proteomic data, confirming the accuracy and reliability of the MS data.

### Validation of candidate target genes of miR-21

**Figure 6:**
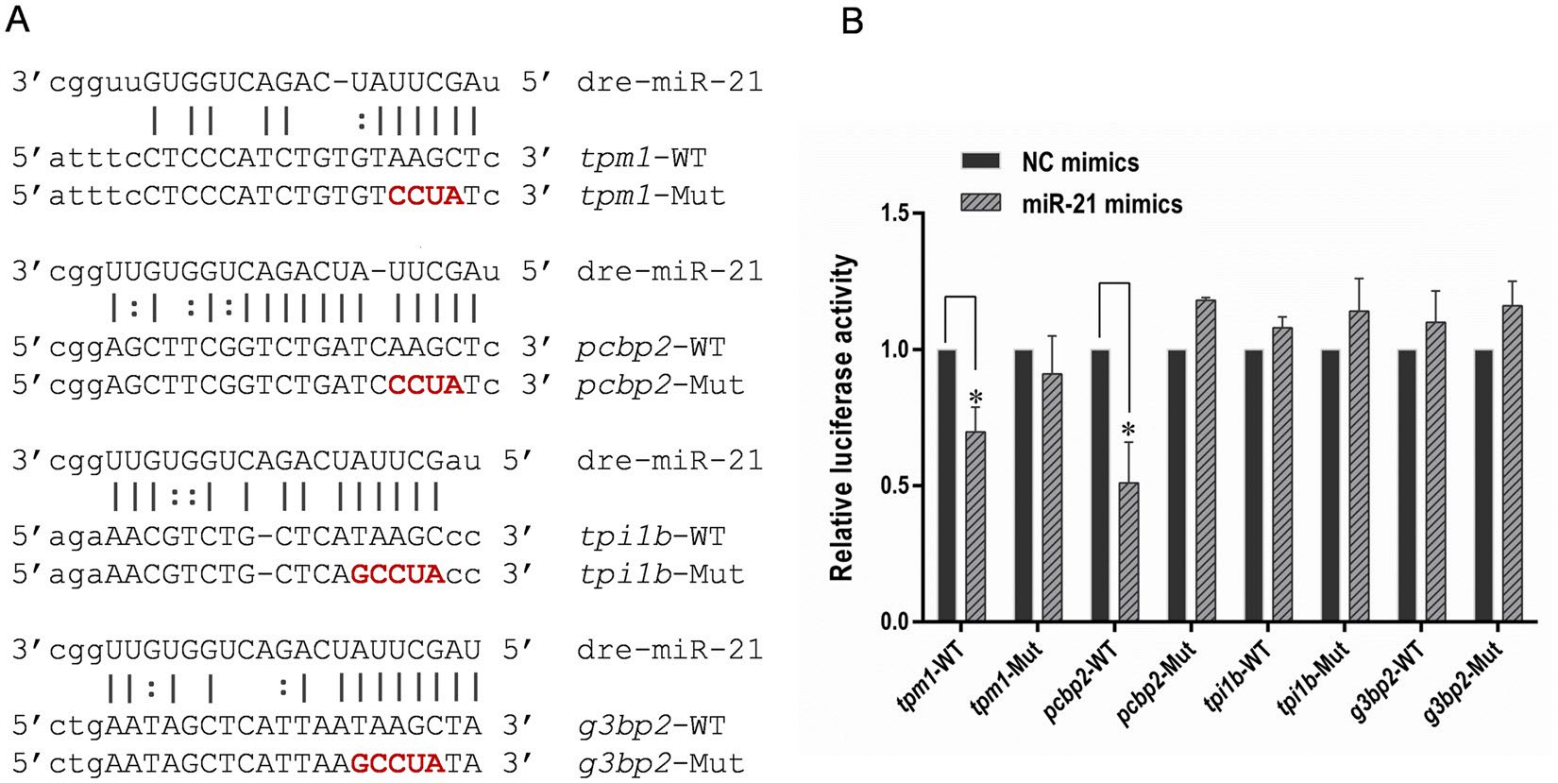
Validation of candidate miR-21 target genes. (**A**) Putative miR-21-binding sites within the 3′-UTRs of *tpm1*, *pcbp2*, *tpi1b* and *g3bp2* genes. Perfect matches are indicated by a line, and G:U pairs by a colon. Nucleotides mutated for the reporter gene assays are underlined. (**B**) Activity of luciferase reporters containing wild type (WT) or mutated (Mut) putative miR-21 target sites in the 3′-UTRs of *tpm1*, *pcbp2*, *tpi1b* and *g3bp2*. The relative luciferase activities normalized to corresponding transfections with NC mimics are shown. Data are shown as the mean ± SD of three replicates and are representative of three independent experiments. *P < 0.05.

Four candidate targets of miR-21 (*g3bp2*, *tpi1b*, *pcbp2* and *tpm1*) were selected, and luciferase assays were performed to determine whether miR-21 directly regulates these potential targets. The 3′-UTRs were PCR-amplified and cloned downstream of the luciferase ORF in the psi-CHECK2 vector. The constructs were co-transfected with miR-21 mimics into HEK293T cells. Significantly decreased luciferase activity was detected in 293 T cells co-transfected with miR-21 mimics and psi-CHECK2-*tpm1*-WT or psi-CHECK2-*pcbp2*-WT (Fig. 6B). By comparison, no significant changes were observed in the luciferase activity in cells co-transfected with the psi-CHECK2 vectors harbouring the 3′-UTR of the other two genes and the miR-21 mimics. Moreover, when the sites complementary to the seed region in the 3′-UTR of miR-21 were mutated, the luciferase activities of these four genes were not affected (Fig. 6). In summary, our results indicate that *tpm1* and *pcbp2* are direct targets of miR-21, while the other two genes may be indirectly regulated by miR-21.

## Discussion

In zebrafish, miR-21 is specifically expressed in the heart and brain and has been shown to be a critical regulator of cardiac valvulogenesis during embryonic development^7, 26^. It can exert its regulatory function by targeting *pdcd4*, *spry2* and *ptenb* ^7, 26^. In the present study, we carried out a loss-of-function experiment by inhibiting the expression of miR-21 in zebrafish embryos. The results showed that knocking down miR-21 resulted in a failure of cardiac looping, which is consistent with previous studies^7, 26^. Then, we performed a TMT-based quantitative proteomic analysis together with bioinformatics and luciferase reporter assays to identify the protein targets of miR-21 in zebrafish embryos. Using this approach, we determined that 251 proteins were differentially regulated after miR-21 inhibition (log2 fold change ≥0.27 or ≤−0.27, CV < 30%). Among these DEPs, 49 were upregulated and 202 were downregulated after miR-21 knockdown (Table S2).

miRNAs are generally believed to have universal effects on gene expression regulation by inducing the degradation of target mRNA or suppressing the translation process^27^. However, increasing numbers of studies have shown that instead of solely exerting a negative regulatory function, miRNAs may also upregulate their targets expression under certain conditions^28–30^. Additionally, miRNAs may oscillate between stimulation and repression in response to specific cellular conditions, RNA sequence context and the presence of cofactors^31^. Thus, proteins which were upregulated after miR-21 inhibition are candidate targets of miR-21. Alternatively, proteins with decreased expression after miR-21 knockdown may either be potential targets or may be downregulated due to indirect effects caused by miR-21. For instance, the downregulated proteins may be downstream factors of another miR-21 target, which participates in the same pathway as the miR-21 target. Several target genes of miR-21 have previously been identified in humans, including *pdcd4* ^14, 15^, *pten* ^16^, *tpm1* ^17^, *spry2* ^18^ and *stat3* ^25^. miR-21 is highly conserved between humans and zebrafish, *pdcd4*, *spry2* and *ptenb* are also confirmed targets of miR-21 in zebrafish^7, 26^. However, only Ptenb was detected in our proteomic analysis (upregulated by 1.28-fold) (Table S2). Pdcd4 and Spry2 were not quantitated, which may be due to the limitations of MS analysis. The upregulation of Ptenb after miR-21 inhibition is consistent with the notion that miRNA may suppress the expression of its target mRNA. A PPI network was generated using the 251 DEPs and the 3 reported miR-21 targets. The results showed that 125 of the 251 DEPs were involved in a PPI network (Fig. S3), implying that these DEPs tend to be functionally associated with each other. Intriguingly, of the three validated miR-21 targets, Spry2 and Ptenb are also associated with these DEPs. Most of these PPIs are putative due to the limitation of the experimentally validated PPI databases in zebrafish. For example, the putative homologs of these proteins have been found to interact or have been reported to associate with each other in curated databases in other species. However, the PPI information will allow us to better understand the functional mechanisms of miR-21.

Hundreds of proteins have been identified to be miR-21-regulated proteins in zebrafish embryos, which are attributed to the priority of the high-throughput MS analysis. The GO classification and pathway analysis revealed that miR-21 may regulate various aspects of the cellular function (Fig. 3), and the miR-21-regulated proteins participate in many different pathways (Table S3). Sixteen proteins were predicted to be miR-21 targets in zebrafish by at least two types of software (Table S4). Of the 16 proteins, Kat7, MurC, Brd9, Tpi1b, MGC174082, Tpm1, Vaspb, G3bp2, Banf1 and Pcbp2 were upregulated at the mRNA level (Fig. 4), indicating that the inhibition effect of miR-21 was weakened due to the knockdown of miR-21, implying that these proteins are potential targets of miR-21. However, only *tpi1b*, *vaspb*, *banf1* and *pcbp2* were upregulated at both the mRNA and protein level, while the other 6 proteins were downregulated at the protein level, suggesting that other post-transcriptional regulatory mechanisms may exist. Four candidates of biological interest (*g3bp2*, *tpi1b*, *pcbp2* and *tpm1*) were selected from the 16 DEPs for luciferase assays. The results showed that overexpression of miR-21 significantly decreased the luciferase activity of the reporter vector containing the 3′-UTRs of *tpm1* and *pcbp2* (Fig. 6).

Tropomyosin (Tpm) proteins play critical roles in modulating muscle contractions^32^. Four *tpm* genes (*tpm1*–*4*) have been identified in vertebrates, and *tpm1* is the only one which has been found to play important roles in cardiac muscle function^33, 34^. Lack of either Tpm1 or Tpm4 is responsible for heartbeat failure^35, 36^. Interestingly, TPM1 is also an miR-21 target in human cancer cells^17^, implying that functional interaction of miR-21 and its targets appears to be conserved in both development and cancer biology. However, the protein expression of Tpm1 was detected to be significantly downregulated after miR-21 suppression by both proteomics and WB. Thus, Tpm1 protein expression may be affected by post-transcriptional regulatory mechanisms, which remain to be uncovered.

Another identified miR-21 target, *pcbp2*, was upregulated at both the mRNA and protein levels after miR-21 inhibition. Poly (C)-binding proteins (PCBPs) are a family of RNA-binding proteins which specifically interact with single-stranded poly (C) regions. PCBP family proteins are involved in various types of posttranscriptional regulation, including mRNA stabilization^37–39^, translational silencing^40, 41^ and translational enhancement^38, 42^. In human cells, PCBP2 is associated with Dicer, and it can bind to miRNA precursors and affect the processing of miRNA precursors^39^, but it can also be targeted by miRNA^43^. In the present study, Pcbp2 associated with 8 proteins in the PPI network (Fig. S3), all of which are nucleic acid binding proteins. Thus, miR-21 may modulate the expression of critical genes in valvulogenesis or other developmental processes by targeting Pcbp2.

## Conclusions

This study is the first to explore the function of miR-21 in zebrafish embryos using a quantitative proteomic approach. Hundreds of proteins which are involved in various aspects of cellular processes were dysregulated after miR-21 inhibition, suggesting that miR-21 is multi-functional. Two genes, *tpm1* and *pcbp2*, were verified to be novel targets of miR-21. Additional miR-21 targets should be identified with the same approach in subsequent studies, allowing a better understanding of the molecular basis of miR-21-mediated regulation of valvulogenesis. The systematic identification of miR-21-regulated proteins provides a valuable source for further in-depth studies of the exact regulatory mechanisms of miR-21 in cardiac valvulogenesis.

## Experimental Procedures

### Zebrafish

Zebrafish (*Danio rerio*, wild type AB line) were raised using standard methods. Embryos were obtained by artificial insemination of adult male and female fish. This study was approved by the Animal Ethics Committee of the Institute of Hydrobiology, Chinese Academy of Sciences. All experiments were performed in accordance with relevant guidelines and regulations.

### MO-mediated Knockdown

MOs were purchased from Gene Tools (Philomath, OR, USA). The MOs used in this study were also used by Banjo and colleagues^7^. Approximately 1 nl of the MOs was injected into the yolk of single-cell embryos. The MO sequences, injection concentrations and doses are listed in Table S5.

### Protein Extraction and Digestion

At 55 hpf, approximately 200 zebrafish larvae pooled from 5 independent MO injection experiments were collected and washed with PBS buffer. 500 µl RIPA lysis buffer (Strong) (Beyotime Institute of Biotechnology, Nanjing, China) containing 1× protease inhibitor cocktail (Thermo Scientific, Waltham, MA, USA) was added to each sample, and then the samples were disrupted by intermittent sonic oscillation (5 s on, 5 s off) for 5 min on ice. Next, the lysates were incubated on a shaker for 30 min at 4 °C. Cellular debris were removed by centrifugation at 12,000 × g for 1 h at 4 °C. Four-fold of ice-cold acetone was added into the supernatant. The supernatant was acetone-precipitated at −20 °C overnight and then centrifuged at 12,000 × g for 30 min at 4 °C. Protein concentrations were determined using the BCA protein assay kit (Thermo Scientific). Part of the protein samples were aliquoted and stored in −80 °C for the western blot (WB) assay. The remaining samples were subjected to trypsin digestion according to Xiong *et al*.^44^. After digestion, a Strata X-C18 SPE column (Phenomenex, Torrance, CA, USA) was used for desalting.

### TMT Labeling

Peptides from two independent biological replicates were vacuum-dried and reconstituted in 0.5 M TEAB, then TMT labelling was performed using the 6-plex TMT kit (Thermo Scientific) according to the manufacturer’s protocol (the labelling reagents 126, 127, 130 and 131 were used). The labelled peptide mixtures were desalted, vacuum-dried and reconstituted in 10% formic acid (FA).

### LC-MS/MS Analysis

Peptide fractionation was performed using high-pH reverse-phase HPLC as previously described^45^. Briefly, peptides were loaded onto an Agilent 300Extend C18 column (4.6 mm ID, 250 mm in length, 5 μm particle size) and separated with a gradient of acetonitrile (2% to 60%) in 10 mM ammonium bicarbonate (pH 8.0) into 80 1-min fractions, and then the peptides were combined into 18 fractions and vacuum-dried. Peptides from each fraction were dissolved in 0.1% FA and analysed by online nanoflow LC-MS/MS with an EASY-nLC 1000 UPLC system (Thermo Scientific) coupled with a Q Exactive™ Plus hybrid quadrupole-Orbitrap mass spectrometer (Thermo Scientific). Briefly, the peptides were loaded onto a reversed-phase analytical column (Acclaim PepMap RSLC, Thermo Scientific) and eluted at a constant flow rate of 300 nl/min. The gradient was set as follows: 6–20% solvent B (0.1% FA in 98% ACN) for 30 min, 20–35% solvent B for 10 min, 35–80% solvent B for 3 min and then 80% solvent B for 4 min. The separated peptides were directly ionized and sprayed into a QE mass spectrometer by a nanospray ion source. Parameters for MS detection were set as follows: the resolution for intact peptides was 70,000; the normalized collision energy (NCE) was 30; and the resolution for ion fragments was 17,500. In the MS survey scan, we use a data-dependent mode with an automatic alteration (1 MS scan followed by 20 MS/MS scans) for the top 20 precursor ions over a ion count of 3 × 10^4^ with 15 s dynamic exclusion. The electrospray voltage was set to 1.8 kV. Automatic gain control was applied to prevent overfilling of the orbitrap. 5 × 10^4^ ions were accumulated for the generation of MS/MS spectra. For the MS scans, the m/z scan range was 350 to 1600 Da, and the fixed first mass was set to 100 m/z.

### Database Search

Peak list files were generated from the raw data using Proteome Discoverer software (Thermo Scientific, v. 1.3.0.339). The generated MS/MS data were processed using the Mascot search engine (v.2.3.0). The zebrafish protein database used for MS/MS searches was downloaded from Uniprot (http://www.uniprot.org/proteomes, 43,095 entries, released 01-02-2016). The parameters for the database search were set as follows: up to 3 missed cleavages were allowed for trypsin; the precursor charge states were set from 1–5; the precursor ion mass tolerance was 10 ppm; the fragment ion mass tolerance was 0.02 Da; fixed modification was set for carbamidomethylation (C), TMT-6plex (K) and TMT-6plex (peptide N-term); and variable modification was set for Oxidation (M). A minimum number of two peptides per protein was required. The FDR at the peptide level was set to 1%, and the minimum peptide score was set to 13.0. Only unique peptides were used for protein quantification.

### qRT-PCR

At 55 hpf, approximately 100 zebrafish larvae pooled from 5 independent MO injection experiments were collected and total RNA was extracted using Trizol (Invitrogen, Gaithersburg, MD, USA). The TaqMan small RNA Assays System was used to detect the expression of mature miR-21 as previously described^26^. Briefly, cDNA was obtained using the TaqMan MicroRNA Reverse Transcription Kit (4366596, Applied Biosystems). qRT-PCR was performed using the RNA assay kit for miR-21 (4440886, Applied Biosystems). *U6* small nucleolar RNA (snRNA) was used as the endogenous control gene. For the predicted miR-21 target genes, total RNA was reverse transcribed into first-strand cDNA using cDNA reverse transcription kit (Invitrogen). mRNA expression level was measured using the LightCycler 480 SYBR Green I Master (Roche Diagnostics, Mannheim, Germany), and *gapdh* was used as the endogenous control. qRT-PCR was performed using the LightCycler 480 Real-Time PCR System (Roche). Triplicate technical replicates were performed for each sample. Results are displayed as means ± S.D. and were compared using a two-tailed Student’s t-test (*P* < 0.05). Primers used for qRT-PCR are shown in Table S5.

### Western Blotting (WB)

WB was performed as previously described^44^. In brief, 10 μg protein extracted from the embryos of both the miR-21 KD and NC groups were separated by 12% SDS-PAGE and transferred to polyvinylidene fluoride (PVDF) membranes (GE Healthcare Waukesha, WI, USA). Then, the PVDF membranes were blocked with 5% nonfat milk and incubated overnight with zebrafish-specific antibodies (Nanog, Pcbp2, Tpm1, Kat7 and Gapdh, 1:1000 dilution) (Abclonal Technology, Wuhan, China), followed by incubation with peroxidase-conjugated anti-rabbit IgG (Abcam, Cambridge, MA, USA) for 1 h (1:3000 dilution) at room temperature. SuperSignal® West Pico Chemiluminescent Substrate (Thermo Scientific) was used to detect the chemiluminescence, and the grey-scale of the protein bands was recorded using ImageQuant TL (GE Healthcare). The intensity of the target protein bands was analysed by ImageJ (National Institutes of Health, Bethesda, MD, USA). Anti-Nanog, Pcbp2, Tpm1, Kat7 and Gapdh polyclonal antibodies were produced and purified via affinity chromatography by Abmart Co. (Shanghai, China). The specificity of the generated antibodies was determined by the manufacturer using ELISA and Western blotting. All WB were performed in three independent experiments.

### Bioinformatics Analysis

The potential targets of miR-21 were predicted using the algorithms of TargetScanFish (http://www.targetscan.org/fish_62/), miRecords (http://c1.accurascience.com/miRecords/), rnahybrid (http://bibiserv.techfak.uni-bielefeld.de/rnahybrid), miRNA viewer (http://cbio.mskcc.org/cgi-bin/mirnaviewer/mirnaviewer4.pl), miRNAMap (http://mirnamap.mbc.nctu.edu.tw/) and microcosm (http://www.ebi.ac.uk/enright-srv/microcosm/htdocs/targets/v5/).

Functional classification by a KEGG analysis of the DEPs was performed using PANTHER^46^. STRING^47^ was used to generate the protein-protein interaction (PPI) network of the DEPs with default settings, except that the organism was set to ‘*Danio rerio*’. PPI networks were visualized with Cytoscape v3.1.0^48^.

### Luciferase Assays

Luciferase assays were performed as previously described with slight modifications^25^. We predicted the 3′-UTR seed sequences of the four candidate targets (*g3bp2*, *tpi1b*, *pcbp2 and tpm1*) using the algorithms of miRrecords. The 3′-UTR fragments of these four genes were PCR-amplified from the cDNA of zebrafish embryos and cloned into a psi-CHECK2 vector (Promega). Primers for 3′-UTR amplification are listed in Table S5. The mutated plasmids psi-CHECK2-*g3bp2*-Mut (the TAAGC sequence in the complementary site for the seed region of miR-21 was mutated to GCCUA), psi-CHECK2-*tpi1b*-Mut (TAAGC to GCCUA), psi-CHECK2- *pcbp2*-Mut (AAGC to CCUA) and psi-CHECK2-*tpm1*-Mut (AAGC to CCUA) were generated from the psi-CHECK2 vector containing the wild type 3′-UTR using a QuickChange kit (Stratagene, La Jolla, CA, USA). All psi-CHECK2 constructs were confirmed by DNA sequence analysis. HEK293T cells were transfected with psi-CHECK2 vectors harbouring the wild type or mutated miR-21-binding site and the miR-21 mimics. Luciferase activity was measured 48 h after transfection using a Dual Luciferase Reporter Assay kit (Promega). All data represent mean values ± S.D. of three independent experiments.

### Statistics

Statistical significance between the experimental groups was analysed by Student’s t-test. *P* < 0.05 was considered to be statistically significant.

## Electronic supplementary material

Dataset 1

Dataset 2

Dataset 3

Dataset 4

Dataset 5

Dataset 6
