# Supplementary material for: Quantitative Proteomics Analysis Reveals Novel Targets of miR-21 in Zebrafish Embryos
Source: Sci Rep. 2017 Jun 22;7:4022. doi: 10.1038/s41598-017-04166-x (PMC5481331; doi:10.1038/s41598-017-04166-x)

**Quantitative Proteomics Analysis Reveals Novel Targets of miR-21 in Zebrafish Embryos.**

Ying Wu^1,2^, Qi-Yong Lou ^1^, Feng Ge^1^, Qian Xiong^1^*

^1^Key Laboratory of Algal Biology, Institute of Hydrobiology, Chinese Academy of Sciences, Wuhan 430072, China

^2^ Graduate University of Chinese Academy of Sciences, Beijing 100049, China

*To whom correspondence should be addressed: Qian Xiong, Institute of Hydrobiology, Chinese Academy of Sciences, E-mail: xiongqian@ihb.ac.cn. Phone/Fax: +86-27-68780730

**Supplementary Figures and Legends**

**Figure S1. Overview of the proteomic results.** (A) Mass errors were determined for all identified peptides. (B) Identified peptide distribution.

**
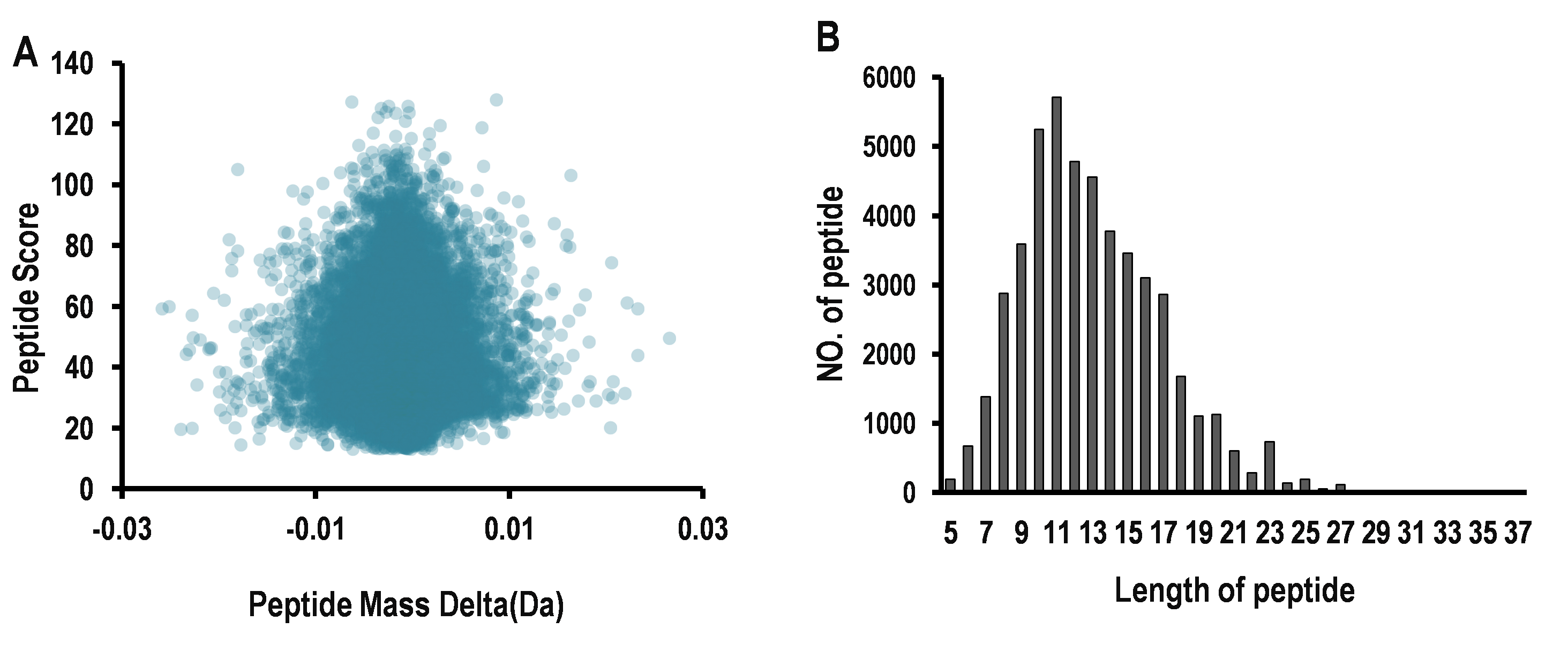
**

**Figure S2. Histogram and Scatterplot showing the quantitative proteomics data. (A)** Histogram showing the frequency distribution of the fold changes of all the quantified proteins. **(B)** Scatterplot of the relationship between proteins quantified in both biological replicates. Red plot indicates differentially expressed proteins and blue plot indicates non-differentially expressed proteins. Protein ratios were log2-transformed and fold change denotes the miR-21-KD/NC ratio.

**
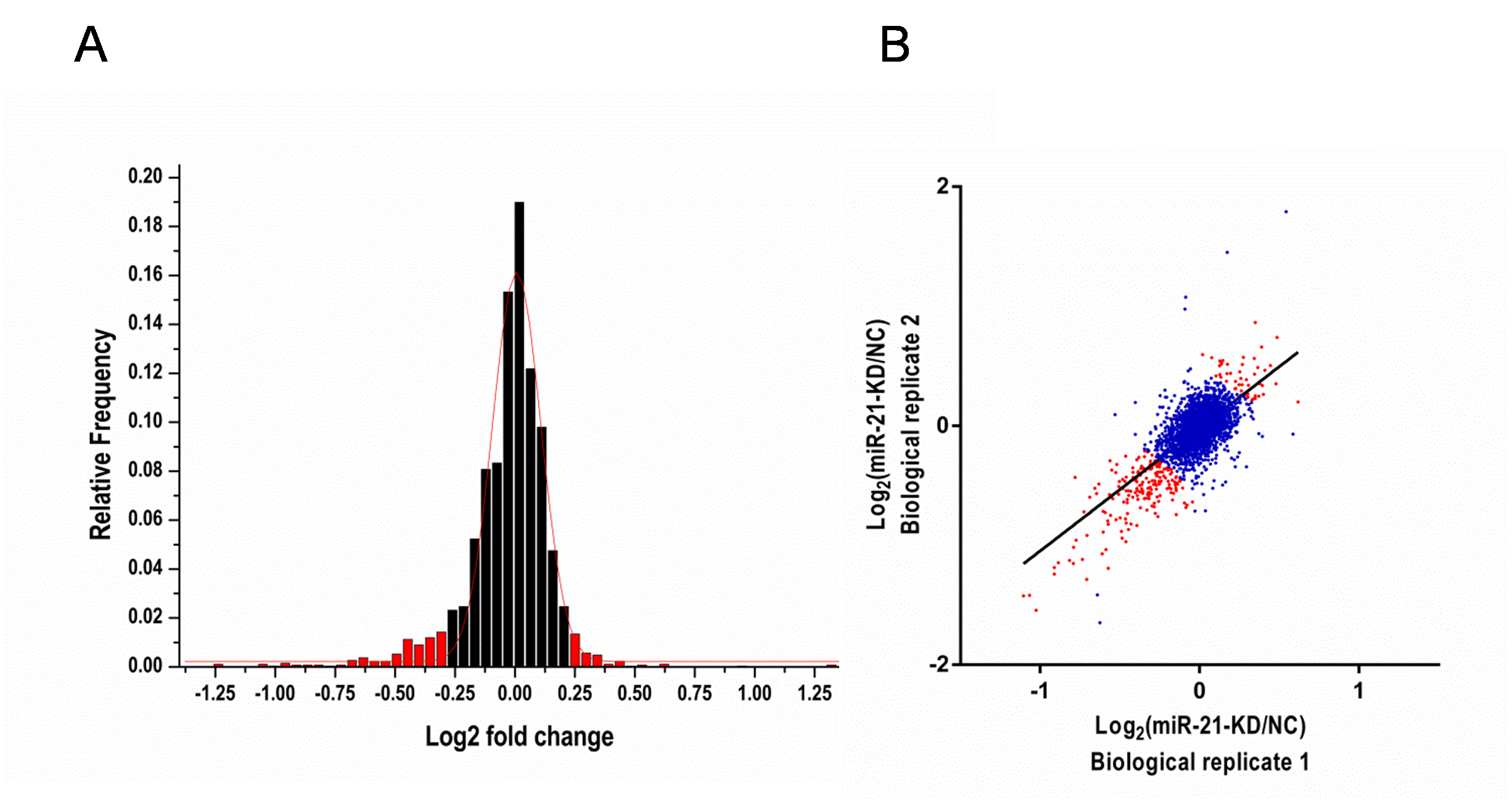
**

**Figure** **S3.** The protein-protein interaction analysis of the DEPs. The PPI network for miR-21-regulated proteins was constructed by searching against the STRING database v10.0 with default settings except that organism was set to “Danio rerio”. 125 of the 251 DEPs are involved in this network.


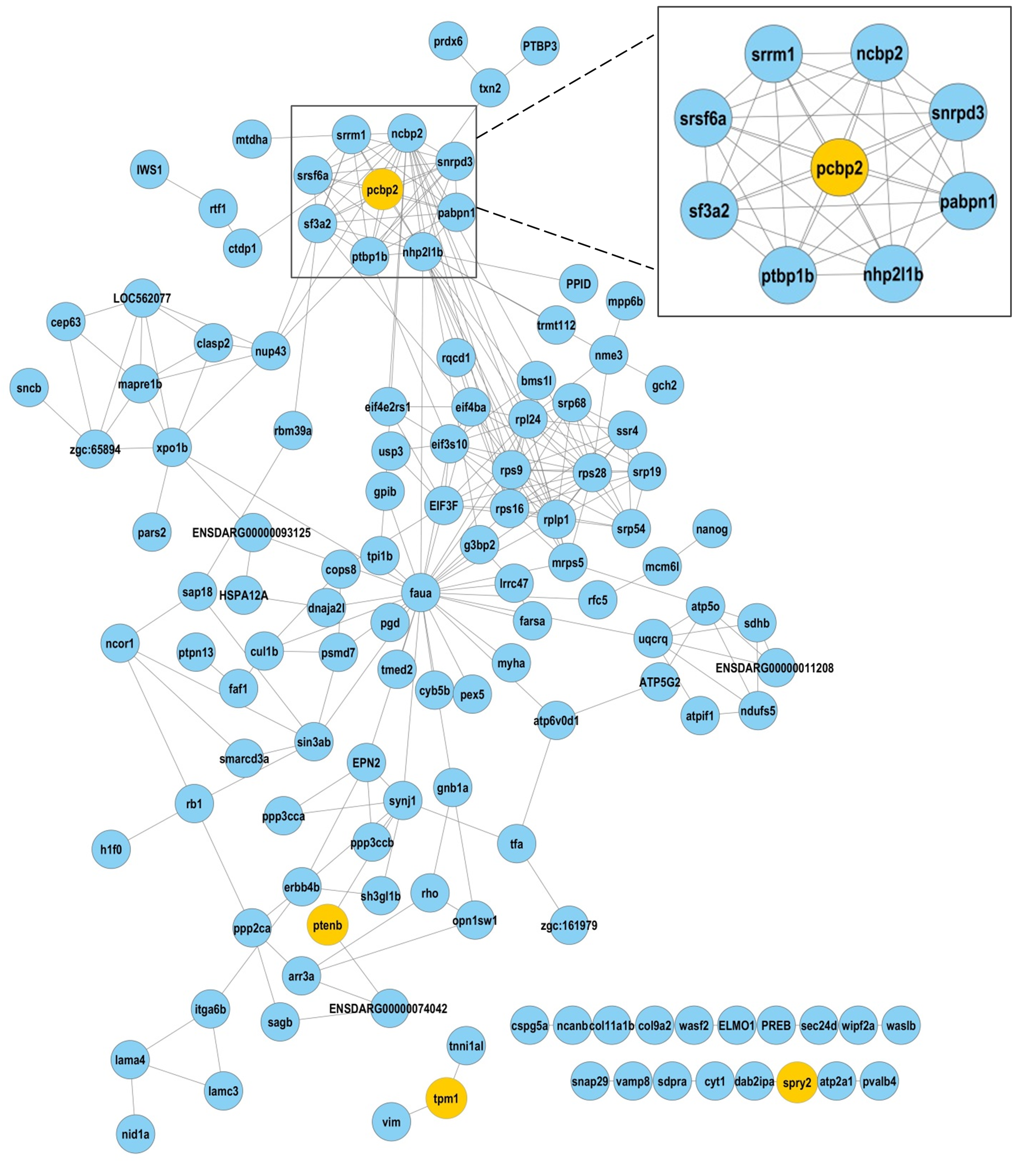


**Figure** **S4. Full image of the Western blots in Figure 5.**

Gapdh 36kD


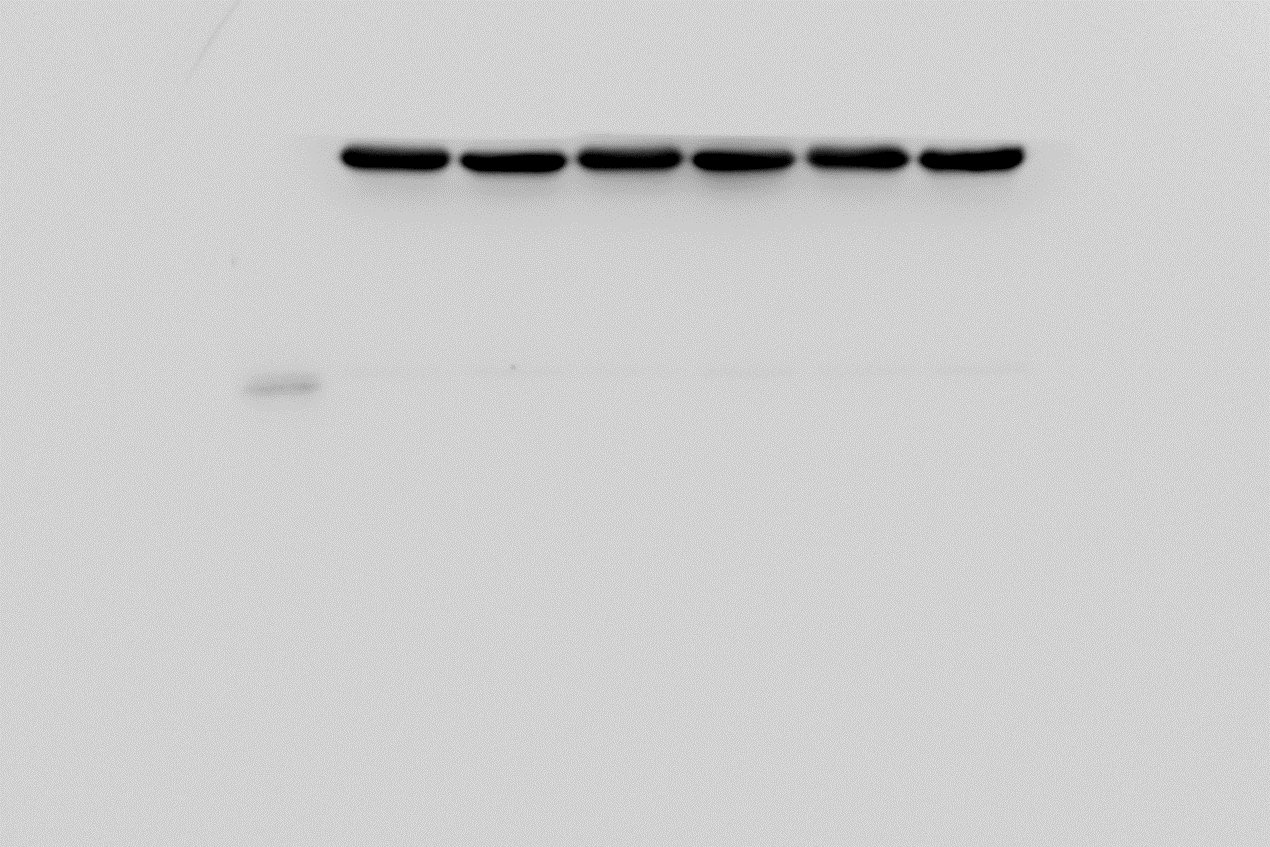


Kat7 66kD


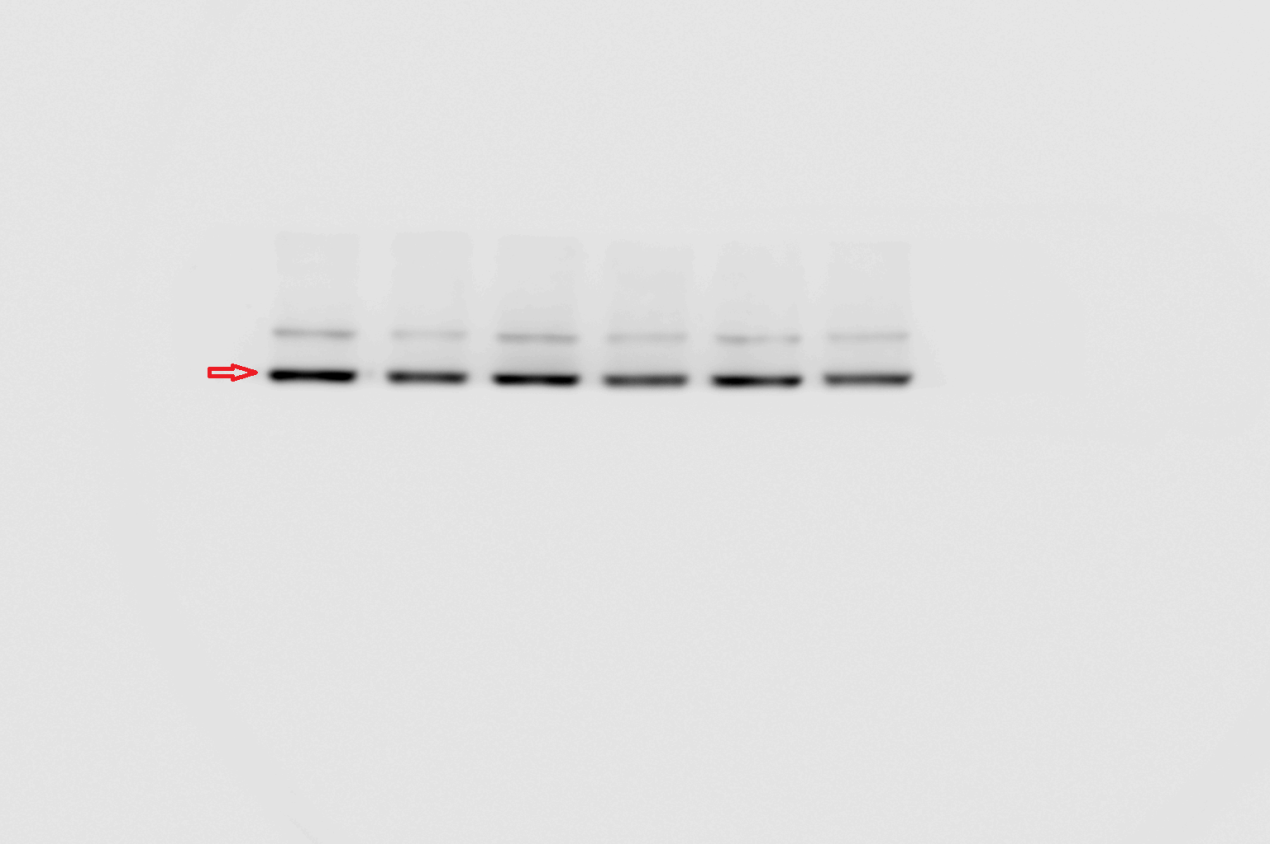


Nanog 42kD


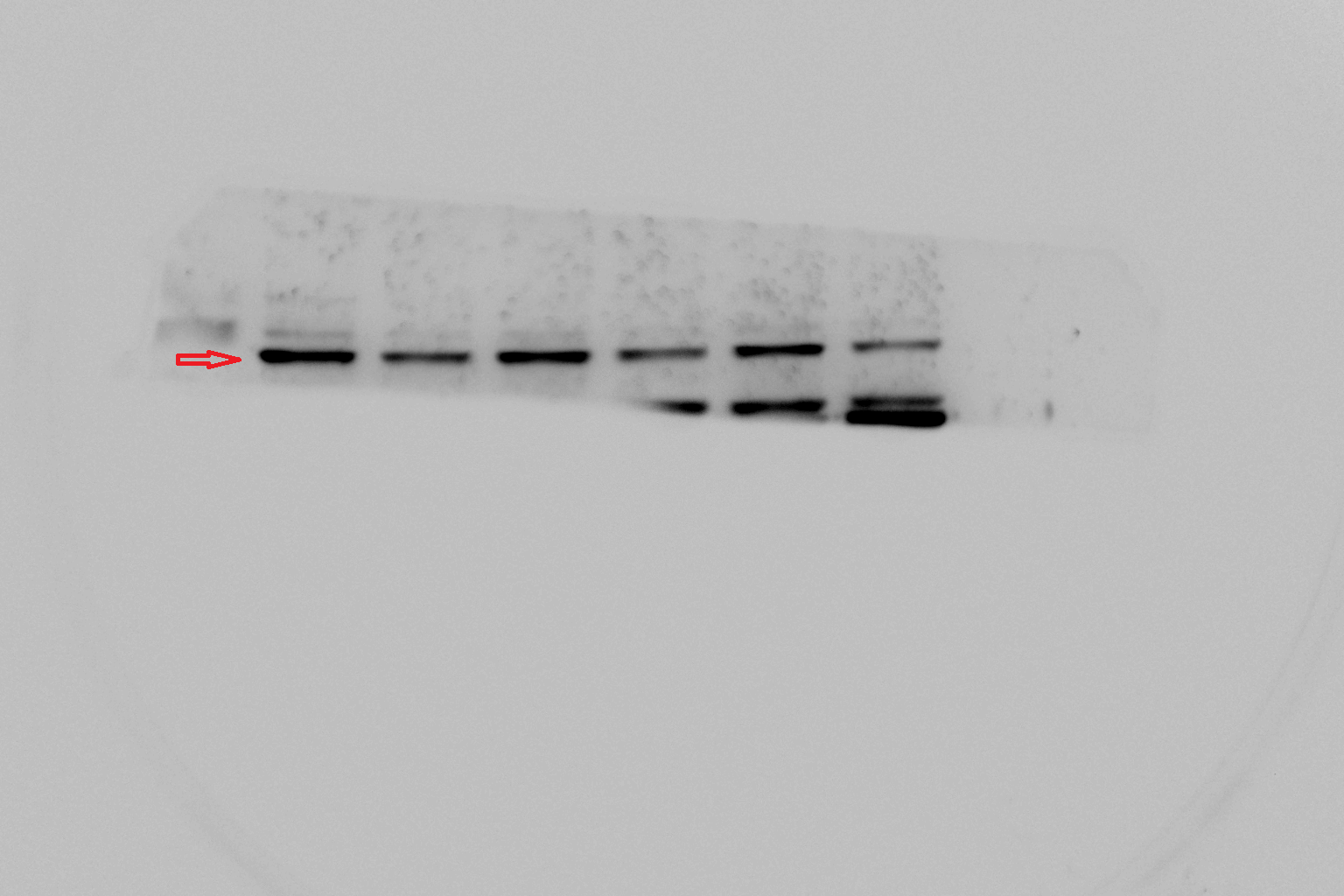


PCBP2 33kD


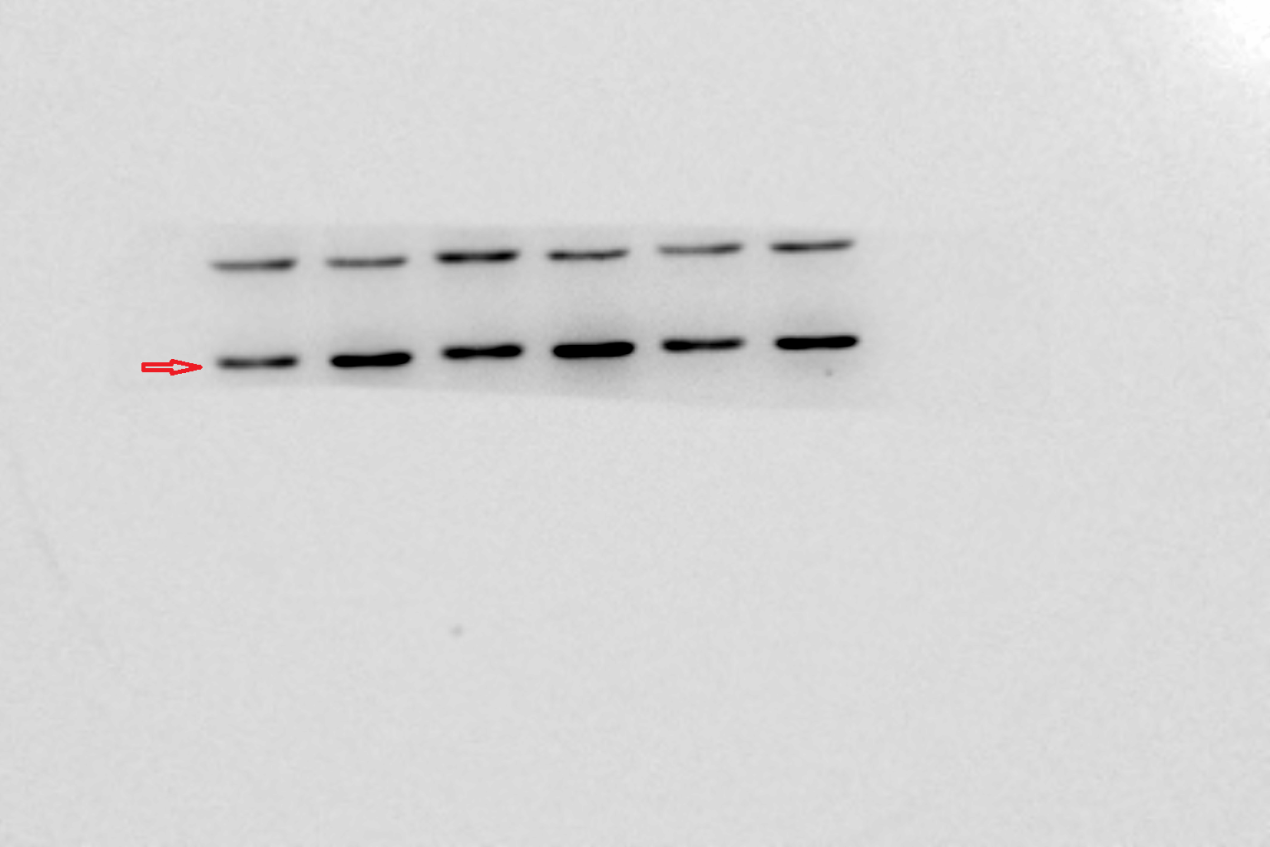


TPM1 33kD


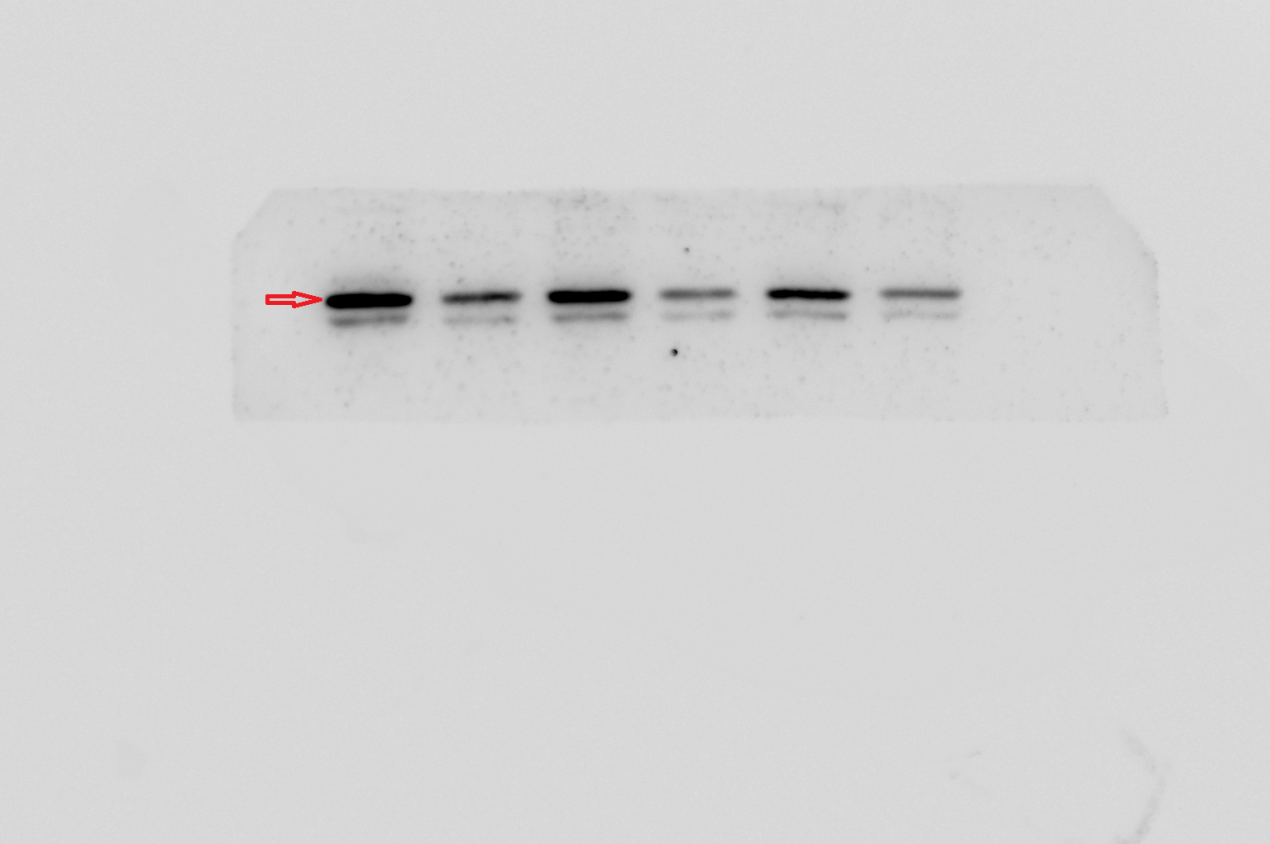

Supplement: Supplementary file 6 — Dataset 6 [file 41598_2017_4166_MOESM6_ESM.doc]
